# Mycobacterial disease in renal allograft recipients

**DOI:** 10.12861/jrip.2013.26

**Published:** 2013-06-01

**Authors:** Mohammad-Reza Ardalan

**Affiliations:** ^1^Chronic Kidney Disease Research Center, Tabriz University of Medical Sciences, Tabriz, Iran

**Keywords:** *Mycobacterium tuberculosis*, Solid organ transplant, Immunosuppression

**Implication for health policy/practice/research/medical education:**

Solid organ transplant recipients have impaired cell-mediated immunity, and are at increased risk of mycobacterial infection.* Mycobacterium tuberculosis* infection (TB) has a high mortality rate among this population. The diagnosis of tuberculosis in solid organ transplant recipients is a big challenge and needs rapid and accurate modalities. These patients have 3.8 time greater risk of developing extra-pulmonary TB than general population. High index of suspicion and applying with invasive diagnostic procedure are needed for diagnosis of TB in renal transplanted patients.

Solid organ transplant (SOT) recipients have impaired cell-mediated immunity, and are at increased risk of mycobacterial infection. *Mycobacterium tuberculosis *infection (TB) has a high mortality rate among this population. The cumulative incidence of post-transplant TB in European and American, SOT recipients ranges from 0.35% to 5%. While in developing countries, the incidence is as high as 15% in some areas of high TB endemic (1-3). That is up to 100-fold higher than that observed incidence, in the general population in the respective countries (2,3). Additionally, renal allograft recipients also coming from dialysis that is a hazardous environment to be infected with TB (4).

On the other hand, kidney transplantation is rapidly growing in developing countries and every day a high number of renal transplanted patients entering the society and increasing the number of this special vulnerable population. The diagnosis of tuberculosis in SOT recipients is a big challenge and needs rapid and accurate modalities (5). These patients have 3.8 time greater risk of developing extra-pulmonary TB than general population (6). Up to one-third of these patients present with disseminated TB. Negative tuberculin skin tests (TSTs) and a typical clinical presentation additionally increasing the diagnostic difficulties (5). Most cases occur as a result of reactivation; but when we retrospectively reviewing the medical history of diagnosed patients only 20–25% of them had a positive TST before transplantation (5). Nonspeciﬁc fever and constitutional symptoms could be the only symptoms and invasive biopsy for histologic diagnosis is essential. Tentative anti-tuberculosis treatment should be considered to make the diagnosis in highly suspicious individuals (6,7). The risk of infection is greatest during the early post transplantation period, when the patient receiving higher dosage of immunosuppressive (6), and two-thirds of cases occur in the ﬁrst post-transplant year (5-9). The risk of post-transplant TB profoundly increasing in those recipients who had delayed graft function and received intense immunosuppressive therapies (5).

With the introduction of mycophenolate mofetile and mammalian target of rapamune inhibitors (m-TOR inhibitors), such as sirolimus and other new immunosuppresive medicines, the risk of post-transplant TB is increasing and there are case reports, where TB manifests immediately after replacing the immunosuppressive regimens with stronger ones (10,11) Transplanted patients are also at increased risk of viral infections such as cytomegalovirus. Viral induced cytokine deregulations could compromise the host’s ability and thereby facilitates reactivation of TB (12). Some of renal allograft recipient have chronic liver disease and diabetes mellitus both are risk factors for development of TB. Diabetes by compromising the cell-mediated immunity facilitate the reactivates of latent TB and the incidence of TB among diabetic patients is 1.5–8 times higher than general population (13,14). Furthermore, more experiences are necessary regarding the administration of anti-tuberculosis agents in renal allograft recipients (6). Rifampin by induction of hepatic cytochrome P-450 3A4 enzyme decreases cyclosporine serum level and could lead to rejection. Hyperuricemia and gouty attacks could happen during the first 2 months of pyrazinamide and cyclosporine therapy (6-8).

Leprosy is a chronic granulomatous disease caused by *Mycobacterium leprae, *and mainly affects skin and nerves. Leprosy still is an important infection in developing countries. It is claimed that immunosuppression does not interfere with the development or aggravation of the manifestations of leprosy. Few cases of leprosy have been reported in SOT recipients, but all of them presented as multi-bacillary leprosy (15). We reported a patient who had a history of recurrent bullous skin lesions before transplantation. After renal transplantation he developed generalized symmetric erythematous papules and pathologic study was compatible with multi-bacillary leprosy. Only 15 cases of leprosy have been reported in organ transplant recipients so far (16), and it should be listed in the differential diagnosis of unusual skin manifestations in organ transplant patients. High index of suspicion and applying with invasive diagnostic procedure are needed for diagnosis of leprosy in renal transplanted patients. Although it is still unknown whether or not immunosuppressive affect the natural history of leprosy, special consideration for this diagnosis is also necessary among SOT recipients.

## Author’s Contribution

MRA is the single author of this paper.

## Conflict of interests

None to declare.

## Ethical considerations

Ethical issues (including plagiarism, informed consent, misconduct, double publication and redundancy) have been completely observed by the authors.

## Fund/Support

No financial support by any institution.
